# Mass Spectrometry-Based Lipidomics in Coffee: Linking Lipid Transformation to Flavor Formation and Quality Control

**DOI:** 10.3390/foods15122196

**Published:** 2026-06-18

**Authors:** Yanbing Wang, Xiaoyuan Wang, Ping Du, Xiaogang Liu

**Affiliations:** 1 Faculty of Modern Agricultural Engineering, Kunming University of Science and Technology, Kunming 650500, China; wongyb@126.com; 2 Horticulture Research Institute, Yunnan Academy of Agricultural Sciences, Kunming 650205, China; 3 Research Center for Analysis and Measurement, Kunming University of Science and Technology, Kunming 652094, China; 4 Analytic & Testing Research Center of Yunnan, Kunming 652094, China

**Keywords:** coffee, lipidomics, aroma formation, flavor formation, roasting, storage stability

## Abstract

Mass spectrometry-based lipidomics has created new opportunities to investigate the role of lipids in coffee quality formation and stability across the production chain. Coffee lipids contribute to flavor precursor formation, aroma release, mouthfeel, and storage behavior, but their molecular remodeling during maturation, processing, roasting, and storage remains insufficiently integrated. This review summarizes recent progress in lipidomics methodologies relevant to coffee research, with emphasis on sample preparation, mass spectrometry platforms, data analysis, and the strengths and limitations of current lipid annotation strategies. It further examines how lipid profiles change during bean maturation, how they differ among coffee species and varieties, and how they are reshaped by postharvest processing, roasting, and storage. However, it is important to note that most of these associations are currently correlational rather than causal; direct evidence linking specific lipid species to particular sensory attributes remains limited. Existing studies suggest that lipid composition, rather than total lipid content alone, is more informative for understanding coffee quality differences and for identifying candidate markers associated with origin, processing method, roasting degree, and storage conditions. In particular, alterations in glycerolipids, glycerophospholipids, fatty acids, diterpenes, and other minor lipid constituents are increasingly associated with lipid oxidation, thermal degradation, and flavor-related transformations in coffee. However, current evidence is still limited by incomplete structural annotation, isomeric ambiguity, platform dependence, and the frequent gap between statistical discrimination and mechanistic validation. Future work integrating high-resolution mass spectrometry, ion mobility, targeted quantification, stable isotope tracing, sensory analysis, and multi-omics approaches will be essential to improve marker reliability and to clarify the functional roles of coffee lipids. Overall, lipidomics provides a promising framework for linking molecular composition with coffee quality control, traceability, and process optimization, although substantial work is still needed to establish mechanistic links to flavor formation.

## 1. Introduction to Coffee Lipids

Coffee is one of the most widely consumed beverages in the world and a major agricultural commodity with substantial economic and cultural importance [1]. Its commercial value depends not only on yield and origin but also on sensory quality, processing stability, and product authenticity [2,3]. The distinctive aroma, flavor, body, and aftertaste of coffee are generated through the interaction of multiple chemical classes, including carbohydrates, proteins, phenolic compounds, alkaloids, and lipids [4,5,6,7]. Among these, lipids are particularly important because they contribute to flavor precursor formation, support aroma release during roasting and brewing, influence mouthfeel and crema stability, and affect storage behavior [3,8,9]. As a result, coffee lipids are increasingly recognized as key determinants of both quality formation and quality preservation.

Coffee beans contain a complex lipid fraction dominated by triacylglycerols, together with phospholipids, free fatty acids, diterpene esters, sterols, tocopherols, and minor lipid-derived compounds [10,11,12]. The composition of these lipids is shaped by species, variety, maturity, environmental conditions, postharvest processing, roasting, and storage [8,9]. These factors do not simply change the total amount of lipid present; they also remodel lipid subclasses and molecular species in ways that can alter the chemical pathways leading to aroma and flavor development. In particular, lipid oxidation and thermal degradation during roasting generate reactive intermediates that may participate in secondary reactions and contribute to the formation of volatile compounds [13,14]. During storage, further oxidation and hydrolysis can accelerate quality deterioration and off-flavor development [15,16]. Therefore, understanding how coffee lipids change across the production chain is essential for improving quality control and process optimization.

Traditional methods for analyzing coffee lipids, such as total lipid content, fatty acid composition, acid value, peroxide value, and other oxidation indices, provide useful but limited information [17,18,19,20]. These measurements are valuable for routine assessment, yet they cannot resolve the structural diversity of lipid species, identify subtle remodeling within lipid subclasses, or capture dynamic changes across different processing stages. In contrast, mass spectrometry (MS)-based lipidomics enables comprehensive profiling of lipid molecules at high sensitivity and high throughput. In food science, lipidomics has already been widely applied to studies of meat [21,22,23,24], dairy [25,26,27], seafood [28,29], cereals [30,31], and edible oils [32,33], where it has improved the understanding of oxidation, traceability, adulteration detection, and product stability.

In coffee research, lipidomics is still nascent and fragmented [34,35,36]. Studies have linked lipid changes to bean maturation, species, processing, roasting, and storage, but findings are scattered across platforms and standards. A unified view of how lipid remodeling shapes coffee quality is lacking. Most work relies on relative abundances and multivariate classification, with limited mechanistic validation or cross-study comparability. Thus, a critical assessment of lipidomics’ current capabilities and gaps is needed. This review provides that assessment, focusing on analytical methods, lipid changes from maturation to storage, their impact on aroma, mouthfeel, stability, and authenticity, as well as current limitations and future strategies. We explicitly distinguish between well-supported correlations and preliminary observations, and we highlight the persistent gap between statistical discrimination and validated mechanistic understanding of flavor formation.

Relevant literature was identified through searches of Web of Science, Scopus, PubMed, ScienceDirect, and Google Scholar, supplemented by manual screening of references from relevant publications.

## 2. Mass Spectrometry-Based Lipidomics for Coffee Analysis

**Figure 1:**
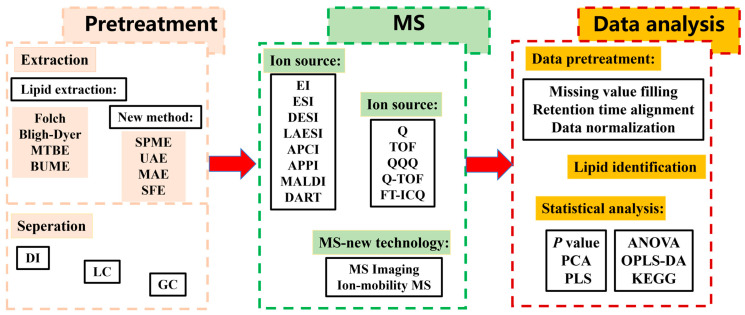
The workflow of MS-based lipidomics. APCI, atmospheric pressure chemical ionization; APPI, atmospheric pressure photoionization; BUME, butanol/methanol; DART, direct analysis in real time; DESI, desorption–electrospray ionization; DI, direct injection; EI, electron ionization; ESI, electrospray ionization; FT-ICQ, Fourier-transform ion cyclotron resonance; GC, gas chromatography; LAESI, laser ablation electrospray ionization; LC, liquid chromatography; MAE, microwave-assisted extraction; MALDI, matrix-assisted laser desorption–ionization; MS, mass spectrum; MTBE, methyl tert-butyl ether; SPME, solid-phase microextraction; SFE, supercritical fluid extraction; TOF, time of flight; Q, quadrupole; QQQ, triple quadrupole; UAE, ultrasonic-assisted extraction.

Due to the essential roles of lipids in biological systems and their close associations with physiological and pathological processes, lipidomics has emerged as an important field for the comprehensive analysis of lipid molecules in specific samples. Its main objective is to characterize the distribution of lipid species, elucidate their biological functions, and reveal the potential mechanisms through which they participate in diverse biological processes [37]. In coffee research, by capturing alterations at the molecular species level, lipidomics can provide a more detailed understanding of coffee quality formation, lipid stability, and potential biomarkers associated with species, origin, and processing conditions. According to the range of lipid metabolites detected and the analytical purpose, lipidomics can generally be divided into targeted lipidomics and untargeted lipidomics [38]. Targeted lipidomics focuses on specific metabolites and achieves accurate quantification through the use of internal standards, thereby providing high specificity and sensitivity for predefined target compounds [39]. In contrast, untargeted lipidomics emphasizes broad coverage, enabling the simultaneous screening of known lipid metabolites and the discovery of novel lipid species with potential biological functions [40]. A complete MS-based lipidomics workflow generally includes sample collection and preparation, mass spectrometry analysis, data processing, and data analysis (Figure 1). Among these steps, mass spectrometry analysis represents the core stage because it generates large amounts of qualitative and quantitative information on lipid species. At the same time, appropriate sample preparation and careful optimization of data analysis parameters are also indispensable for ensuring data quality and improving the reliability and accuracy of the final results.

### 2.1. Lipid Extraction Strategies

Lipid extraction is a critical preliminary step in lipidomics because extraction efficiency directly affects the comprehensiveness and reliability of subsequent analyses. At present, liquid extraction (LE) remains the most widely used strategy. Because lipid solubility in organic solvents is determined by both hydrophobic fatty acid chains and hydrophilic head groups, such as phosphate or sugar residues, the extraction method must be selected according to the sample matrix and target lipid classes in order to maximize efficiency [41]. Nonpolar solvents, including hexane, petroleum ether, and supercritical carbon dioxide, are generally used for neutral lipids, whereas polar solvents such as methanol and acetonitrile are more suitable for highly polar lipids, including phospholipids [42,43].

For comprehensive lipid profiling, mixed solvent systems containing both polar and nonpolar components, such as methanol, butanol, water, methyl tert-butyl ether, or chloroform, are commonly employed. Classical liquid–liquid extraction methods, including the Folch, Bligh and Dyer, and Matyash methods, as well as their modified versions, remain widely used for biological and food samples because they enable efficient recovery of most lipid classes and generate extracts compatible with downstream analyses. More recently, one-phase extraction approaches based on methanol/t-butyl methyl ether/chloroform or isopropanol have been developed and shown to offer greater convenience and advantages in lipid recovery and analytical coverage [44,45,46].

In addition to conventional LE, several microextraction techniques have been introduced to improve extraction efficiency, especially in targeted lipidomics. For example, dispersive liquid–liquid microextraction (DLLE) and ionic liquid microextraction (ILME) have shown high extraction efficiency in specific applications. Solid-phase extraction (SPE), by contrast, selectively retains target lipids on a stationary phase and is often used alone or in combination with LE to fractionate crude lipid mixtures and enrich trace lipid species. Various stationary phases can be applied in SPE, including silica, C8, C18, and ion-exchange columns. For instance, C18 columns have been used to purify sterols from virgin coconut oil and to enrich lipid oxidation products in meat, fish oil, and milk powder samples [47,48].

Several emerging extraction technologies have also attracted attention in recent years, including solid-phase microextraction (SPME), ultrasound-assisted extraction (UAE), microwave-assisted extraction (MAE), and supercritical fluid extraction (SFE), all of which have shown good performance in efficient lipid extraction [46,49]. Among them, SPME is a non-equilibrium technique based on the partitioning of analytes between the sample matrix and an extraction phase immobilized on fused silica fibers or inner coatings. Owing to its low cost, simplicity, and high reproducibility, SPME has been widely used for enriching short-chain volatile fatty acids [50], polyunsaturated fatty acids [51], trans fatty acids [52], lipid oxidation products, and even total lipids in food and biological matrices [53]. In addition, the QuEChERS method, originally developed for pesticide residue analysis, has recently been extended to the extraction of trace lipid contaminants [54]. Overall, these emerging methods have further improved the efficiency, selectivity, and versatility of lipid extraction in lipidomics studies.

The coffee bean matrix is rich in carbohydrates, proteins, phenolics, and other compounds that may interfere with lipid recovery or ionization during analysis. Therefore, the choice of extraction strategy should be carefully matched to the analytical objective and the target lipid classes. In broad profiling studies, mixed solvent systems are commonly used to recover both neutral and polar lipids, whereas targeted studies may require more selective extraction or enrichment procedures to minimize interference. Although many extraction methods have been developed, no single protocol is universally optimal for all coffee matrices. Differences in bean type, roasting degree, storage status, and moisture content may all affect extraction efficiency and downstream reproducibility, underscoring the need for method optimization on a case-by-case basis. Notably, most published coffee lipidomics studies have adopted protocols developed for plasma or tissues without systematic validation for coffee matrices; future work should include matrix-matched recovery tests.

### 2.2. Mass Spectrometry-Based Lipidomics Platforms

Traditional methods for food lipid analysis, such as the determination of total lipid content, fatty acid composition, acid value, peroxide value, and carbonyl value, mainly provide basic information on lipid content and oxidation status. However, they offer limited insight into lipid composition and structural features. With the continuous development of analytical technologies, mass spectrometry (MS) has become the dominant platform in lipidomics because of its outstanding specificity, sensitivity, dynamic range, and throughput, enabling deeper characterization of lipid composition, structure, and function.

In general, MS-based lipidomics strategies can be divided into two major categories: direct infusion mass spectrometry (DI-MS) and chromatography–mass spectrometry (LC-MS) [55]. DI-MS is attractive because of its high throughput and broad applicability, allowing the detection of hundreds of lipids within a short time and with relatively simple operation [56]. However, complex sample matrices may cause matrix effects that interfere with target detection. To address this limitation, chromatography is often coupled with MS to reduce interference from coexisting components. Gas chromatography (GC) is generally suitable for weakly polar, volatile, and low-molecular-weight lipids such as fatty acids, whereas liquid chromatography (LC) is more appropriate for comprehensive lipid characterization [57].

In addition to DI-MS and LC-MS, ion mobility–mass spectrometry (IM-MS) and mass spectrometry imaging (MSI) have developed rapidly in recent years, further broadening the application of lipidomics in food research. As a separation technology orthogonal to LC, IM-MS is particularly useful for resolving isomeric lipids and improving separation efficiency [58]. MSI, in contrast, enables in situ visualization of the spatial distribution and dynamic changes of lipids, thereby providing a unique perspective for studying lipid heterogeneity in food systems. However, its widespread application is still limited by high instrumental cost, massive data output, and substantial technical demands.

Recent progress in mass spectrometry has involved both methodological innovation and continuous improvement in instrument performance. Different ionization techniques and mass analyzers provide distinct advantages in sensitivity, reproducibility, and mass resolution [59]. For example, FTICR provides the highest mass resolution (>1,000,000 at full width at half maximum) and excellent mass accuracy (<1–2 ppm), although its sensitivity is moderate. Orbitrap instruments also offer very high mass resolution, ranging from 100,000 to 800,000, with mass accuracy below 5 ppm and medium sensitivity. In contrast, triple quadrupole instruments have relatively low mass resolution (approximately 1000) and comparatively poor mass accuracy (100–1500 ppm), but they provide high sensitivity and excellent quantification capability. Q-q-TOF and TOF/TOF-TOF instruments also exhibit high sensitivity, while the linear ion trap provides good sensitivity [60]. Particularly noteworthy is direct analysis in real time (DART), which enables direct, rapid, non-destructive, and in situ analysis and is therefore highly suitable for high-throughput applications [61]. Together, these technological advances are enabling increasingly detailed exploration of lipid characteristics and functions. For coffee specifically, the choice of ionization source is critical: diterpenes (cafestol, kahweol) are poorly ionized by ESI, while roasted coffee matrices demand longer LC gradients to mitigate ion suppression from Maillard reaction products [34].

In coffee lipid research, direct infusion approaches can provide rapid screening and high throughput, but they are often limited by ion suppression and reduced selectivity in complex matrices. Chromatography-coupled mass spectrometry is therefore more commonly used when deeper structural resolution is needed. Liquid chromatography is particularly suitable for separating complex lipid mixtures, while gas chromatography remains useful for fatty acid analysis and other volatile or derivatized components. More advanced platforms, including ion mobility–mass spectrometry and mass spectrometry imaging, have further expanded the analytical toolbox by improving the separation of isomeric species or enabling spatially resolved analysis. These methods are promising for coffee research, but their use remains limited by instrument cost, data complexity, and the need for specialized interpretation. Moreover, several advanced platforms, such as IM-MS, MSI, and DART-MS, have not yet been extensively applied to coffee lipidomics, and their practical value for coffee-specific questions still requires further validation.

### 2.3. Lipidomics Data Processing and Chemometrics

MS-based lipidomics generates large and complex datasets from which information on target lipids or global lipid profiles must be extracted. In food lipid research, these descriptive and discriminative data can be integrated with chemometric tools to establish predictive models for geographical traceability, adulteration detection, food composition analysis, and improved control of harmful lipids and lipid-derived reaction products during food processing [55]. Accordingly, data processing and statistical analysis are indispensable components of lipidomics workflows.

With ongoing improvements in MS resolution and scanning speed, the scale of lipidomics datasets has increased substantially. As a result, the extraction of meaningful information for lipid identification and quantification has become both essential and time-consuming. In general, this process consists of two main stages: data preprocessing and data analysis. During preprocessing, researchers commonly use vendor software such as PeakView and LipidSearch; open-source platforms including Lipid Data Analyzer, XCMS, MZmine, and MS-DIAL; or custom scripts developed in MATLAB or R to perform filtering, peak detection, alignment, and normalization [62,63]. Lipid identification is then achieved by comparing fragmentation patterns with lipid databases. However, accurate annotation in lipidomics remains challenging because many lipid species share the same nominal mass or differ only in subtle structural features, such as chain length, unsaturation position, or sn position. Consequently, reliable identification generally requires the integration of multiple types of evidence, including isotope patterns, MS/MS fragmentation spectra, and chromatographic retention behavior, rather than relying on *m*/*z* matching alone. Commonly used resources include the Lipid Metabolites and Pathways Strategy (Lipid MAPS) structure database, Lipid IMMS Analyzer, Lipid Library, and Lipid Bank. Among them, Lipid MAPS is widely regarded as the most authoritative resource for lipid classification and nomenclature because it provides comprehensive qualitative and quantitative tools and can predict potential lipid species based on *m*/*z* values, MS/MS spectra, or characteristic fragment ions [57]. These resources have greatly improved the efficiency and accuracy of lipid identification and quantification. Nevertheless, in studies of complex matrices such as coffee, many reported lipid markers are still annotated only at the class or molecular species level rather than as fully resolved structural isomers. This limitation should therefore be considered when interpreting candidate biomarkers, since different isomers may differ in abundance, stability, or biological relevance despite appearing similar in conventional analyses.

After preprocessing and identification, appropriate statistical methods are selected according to specific research objectives. In lipidomics, a common strategy is to compare differences between groups, such as control and treatment groups, in order to identify significantly altered lipid molecules. For this purpose, univariate methods such as the *t*-test and analysis of variance (ANOVA) are widely used. The *t*-test is generally applied to comparisons between two groups, whereas ANOVA is suitable for multiple-group comparisons. However, because lipidomics datasets are often highly multidimensional, multivariate data analysis (MVDA) is also essential for evaluating major effects and interactions among variables. Depending on the analytical objective, multivariate approaches can be categorized into exploratory analysis, classification or discriminant analysis, and regression or predictive modeling. Principal component analysis (PCA) and hierarchical cluster analysis (HCA) are among the most commonly used exploratory methods. PCA reduces data dimensionality by removing redundant information and transforming the remaining variation into principal components, whereas HCA classifies samples according to similarity and dissimilarity.

For discriminant analysis, commonly used methods include linear discriminant analysis (LDA), partial least squares discriminant analysis (PLS-DA), and orthogonal partial least squares discriminant analysis (OPLS-DA). In regression and predictive modeling, multiple linear regression (MLR), partial least squares regression (PLS), orthogonal partial least squares regression (OPS), and genetic algorithm (GA)-based methods are frequently employed. Despite their popularity, supervised classification models must be interpreted with caution. Clear separation in score plots does not necessarily indicate biological causality, and overfitting remains a common concern, particularly in studies with limited sample size or insufficient validation. Increasingly, machine learning approaches such as random forests (RFS) and support vector machines (SVMS) are being incorporated to improve predictive modeling and feature selection [55,57,64], although their robustness also depends on careful model validation.

For coffee research, the most informative lipidomics studies are those that integrate comprehensive lipid profiling with rigorous validation strategies. Candidate lipid markers should ideally be confirmed using independent datasets, targeted quantification, or complementary sensory and processing data. In this context, lipidomics should be considered not merely as a descriptive analytical technique but as an integrative framework for linking molecular composition with coffee quality attributes, including origin discrimination, processing evaluation, storage stability, and flavor-related lipid pathways.

Overall, lipidomics provides a powerful analytical foundation for understanding chemical variation across the coffee production chain. However, its interpretative value ultimately depends on methodological rigor and cautious data interpretation. Structural ambiguity, platform-dependent variability, and the frequent gap between statistical association and mechanistic explanation remain key limitations that should be explicitly considered when translating lipidomic findings into practical applications for coffee quality assessment. In coffee lipidomics, three additional caveats deserve emphasis: (i) most reported markers are annotated only at the sum-composition level without regioisomer resolution, yet isomers differ in stability and reactivity; (ii) coffee-specific lipids (e.g., diterpene esters) are poorly covered by standard databases like LipidMAPS; (iii) many studies use small sample sizes (n = 3–6), increasing overfitting risk in multivariate models. Cross-validation and external validation are strongly recommended [57,64].

## 3. Applications of Lipidomics in Coffee Research

**Figure 2:**
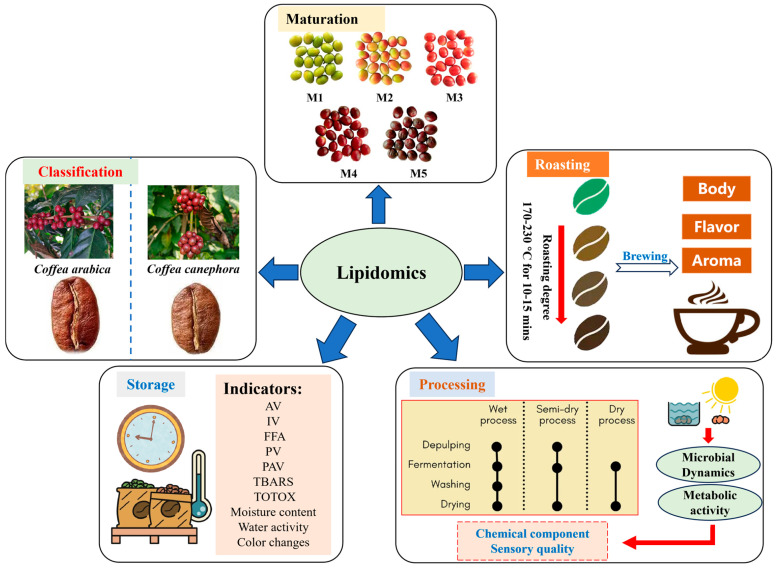
Applications of lipidomics across the coffee production chain.

Lipidomics provides molecular-level insight into lipid remodeling across the coffee production chain, including variety selection, cultivation, maturation, postharvest processing, roasting, and storage (Figure 2). MS-based lipidomics can determine lipid composition and relative abundance in coffee across different varieties [34], geographical origins, maturation stages, processing methods, and roasting degrees, thereby supporting origin traceability, variety identification, process optimization, oxidation monitoring, and authenticity assessment [35]. However, current studies differ substantially in sample type, analytical platform, lipid annotation level, and validation strategy, making direct comparison difficult. To provide a clearer and more critical overview of the available evidence, the main coffee lipidomics studies are summarized in Table 1. This table compares the research area, key lipidomic findings, evidence strength, major limitations, and references for representative studies on coffee maturation, species and variety discrimination, postharvest processing, roasting, and storage.

### 3.1. Lipidomics in Coffee Bean Maturation

Coffee bean maturation is a dynamic developmental process during which the chemical composition of the seed gradually changes in parallel with fruit ripening [8,87]. These changes are reflected not only in external color and texture but also in the accumulation and remodeling of key metabolites that later influence roasting behavior and sensory quality [88]. Among these metabolites, lipids are of particular interest because they contribute to flavor precursor formation, membrane stability, and the physicochemical properties of the bean matrix [89,90]. Understanding how lipid profiles change during maturation is therefore relevant to both coffee quality evaluation and the selection of optimal harvest maturity.

Available studies indicate that maturation is associated with changes in both total lipid content and lipid composition, although the magnitude and direction of change are not always consistent across cultivars and experimental conditions [91,92,93]. In some cases, overripe beans have been reported to contain higher total lipid levels than mature beans [91], whereas other studies found only minor differences across maturity stages [92,93]. These inconsistencies suggest that maturation does not exert a uniform effect on lipid accumulation across all coffee types. Instead, the observed variation may depend on genotype, environmental conditions, fruit development stage, and the analytical methods used for lipid measurement. As a result, total lipid content alone is not sufficient to capture the full extent of maturation-related lipid remodeling.

Lipidomic analyses provide a more detailed view of this process by revealing changes at the subclass and molecular species levels [65,66]. During maturation, glycerolipids and glycerophospholipids appear to be among the most responsive lipid classes, with phosphatidic acid, lysophosphatidic acid, and diacylglycerol frequently emerging as important components in developmental lipid remodeling [65]. Some studies have reported that immature beans contain lower levels of phospholipids and palmitoylated or linoleoylated lipid species than mature or overripe beans, suggesting progressive accumulation or reorganization of membrane-associated lipids during fruit development [66]. Other studies have shown that fatty acid composition changes only modestly, even when total lipid abundance varies more clearly [93]. Taken together, these findings indicate that maturation may have a stronger impact on lipid class distribution and molecular species composition than on bulk lipid quantity.

**Figure 3:**
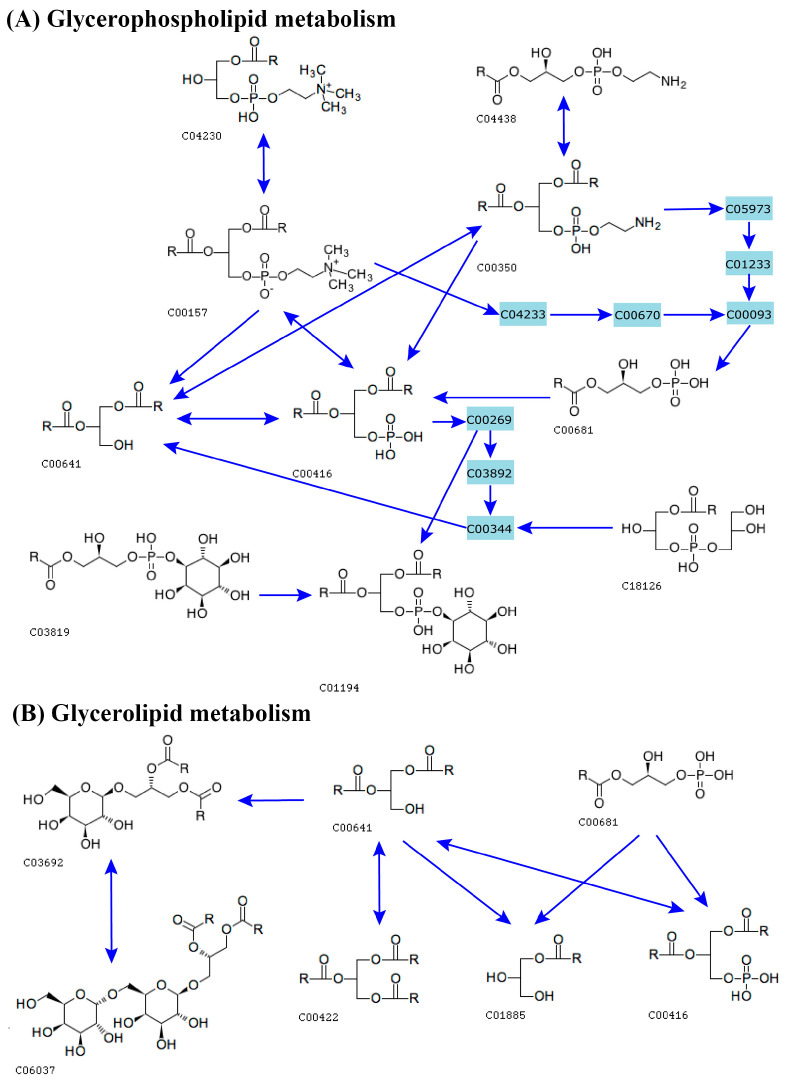
Overview of significant lipid metabolic pathways in coffee during maturation [65]: (**A**) glycerophospholipid metabolism; (**B**) glycerolipid metabolism. The numbers correspond to the ID of each lipid subclass.

From a practical perspective, these maturation-related lipid shifts may be relevant to downstream quality formation. Lipid composition established during bean development can influence the substrate pool available for roasting-related reactions, thereby affecting aroma generation, mouthfeel, and storage behavior [89,90]. At the same time, lipidomic profiles may also provide candidate markers for assessing maturity stage and harvest readiness (Figure 3) [65]. However, current evidence is still limited by differences in sample origin, maturity definitions, and analytical resolution. In many studies, maturity categories are broad, making it difficult to determine which specific developmental transitions drive the observed lipid changes. In addition, most reports remain descriptive and have not directly linked maturation-associated lipid remodeling to sensory outcomes [65,66,91,92,93].

Overall, the current literature suggests that coffee bean maturation is accompanied by meaningful but context-dependent lipid remodeling, particularly in glycerolipid and glycerophospholipid metabolism [65,66]. Lipidomics is well suited to capture these changes at a molecular level and may help clarify how developmental stage shapes the chemical foundation of coffee quality. Future work should use standardized maturity criteria, improved structural annotation, and targeted validation to distinguish robust maturation markers from cultivar-specific variation. At present, no study has directly demonstrated that maturation-induced lipid remodeling causally affects specific sensory attributes of the final brew. The link therefore remains inferential, based on the assumption that precursor pools established during maturation influence roasting reactions.

### 3.2. Lipidomics for Coffee Species and Variety Discrimination

Coffee species and varieties differ in agronomic performance, sensory quality, and commercial value, making reliable discrimination an important issue for both quality control and market authentication [94,95]. In practice, visual assessment alone is often insufficient to distinguish closely related coffee materials, particularly when roasted, ground, or blended products are involved. Lipid composition is therefore attractive as a discriminative feature because it reflects both genetic background and, to some extent, the developmental and physiological characteristics of the bean.

Among the major cultivated coffee species, Arabica beans generally contain about 15% lipids, higher than the approximately 10% typical of Robusta [17]. In addition to this difference in total lipid content, Arabica and Robusta also differ in fatty acid composition. Arabica generally exhibits relatively higher proportions of palmitic acid (16:0), stearic acid (18:0), arachidic acid (20:0), and linolenic acid (18:3), whereas oleic acid (18:1) is usually more abundant in Robusta [67,68]. These compositional differences provide a molecular basis for species discrimination, but they also reflect the broader metabolic divergence between the two species [96]. In addition, the higher lipid content of Arabica may contribute to its generally smoother mouthfeel and more valued sensory profile, although sensory quality cannot be inferred from lipid composition alone.

Lipidomic studies have identified several candidate molecules that may help distinguish Arabica from Robusta [69]. Differences in triacylglycerol species, fatty acid profiles, diterpene esters, and minor lipids such as sterols and tocopherols have all been reported [70,71,72,73,74,75]. In particular, some triacylglycerols and fatty acids appear to be more abundant in one species than the other [70,71], suggesting that specific lipid subclasses may serve as useful chemical markers. However, the utility of these markers depends on analytical resolution, sample origin, and the extent to which environmental factors are controlled. Because coffee lipid profiles are also influenced by altitude, shading, temperature, and other growing conditions [94], species-level discrimination based on lipids should be interpreted within the context of genotype-environment interaction rather than treated as a purely species-specific signal.

Within species, lipidomics may also assist in distinguishing varieties or genetic groups. This is especially relevant for Arabica, where substantial diversity exists among cultivars and breeding materials [77,97,98,99]. Several studies have reported that certain triglycerides or fatty acids can separate accessions or geographic populations, indicating that lipid profiles retain information about genetic background [78,79]. At the same time, these signals may be influenced by maturity stage, postharvest handling, and roasting, which limits their direct transferability across datasets. For this reason, candidate lipid markers for variety discrimination should ideally be validated across independent sample sets and under standardized analytical conditions.

From an applied perspective, lipidomics-based discrimination offers potential value for authenticity testing, origin verification, and the detection of adulteration in coffee products. This is particularly relevant in commercial settings where higher-value Arabica may be mixed with Robusta or where cultivar identity is difficult to confirm by appearance alone [68,69]. Nevertheless, current studies are still largely descriptive and depend heavily on multivariate separation rather than mechanistic explanation. In many cases, the reported markers are useful for classification, but not yet fully established as robust universal identifiers. More systematic validation, especially across multiple origins, harvest years, and processing conditions, will be necessary before lipid markers can be translated into routine authentication workflows.

Overall, lipidomics provides a useful framework for distinguishing coffee species and varieties by capturing genotype-associated differences in lipid metabolism and composition. The most informative features are likely to arise from combinations of triacylglycerols, fatty acids, diterpenes, and minor lipid classes rather than from any single compound. Future studies should emphasize marker stability, cross-cohort reproducibility, and the interaction between genetic and environmental effects in order to improve the reliability of lipid-based classification systems.

### 3.3. Lipidomics in Postharvest Processing of Coffee

After harvesting, coffee cherries remain enclosed within a pectin-rich fruit pulp and must undergo postharvest processing to obtain green beans with a moisture content of approximately 10–12%. The main processing methods include dry, wet, and semi-dry approaches, all of which influence microbial activity, endogenous metabolism, and subsequent chemical transformations in the bean matrix [100]. Among the major coffee constituents, lipids are particularly relevant because they serve as important flavor precursors and contribute to the development of coffee aroma, body, and overall sensory quality. By contrast, compounds such as chlorogenic acids, caffeine, and trigonelline tend to show comparatively smaller variation across processing methods, whereas lipid composition often changes more substantially [101].

Existing studies indicate that processing conditions can affect both total lipid content and the relative abundance of specific lipid classes. For example, Singh et al. [102] reported that small-sized Gayo coffee beans processed by the wet method showed a higher fat content than those processed by the dry method, and Kitzberger et al. [103] similarly observed higher lipid retention in wet-processed small coffee varieties. These findings suggest that processing-associated biochemical or physicochemical changes may contribute to differences in lipid retention or extractable lipid content, although the extent of these effects likely depends on bean type, processing intensity, and local processing conditions. During postharvest processing, lipid remodeling may be influenced not only by the processing method itself but also by fermentation intensity, microbial activity, moisture exposure, oxygen availability, and drying conditions. However, coffee-specific studies integrating lipidomics with microbiota profiling remain limited, and the contribution of fermentation-associated microorganisms to lipid remodeling still requires further validation.

**Figure 4:**
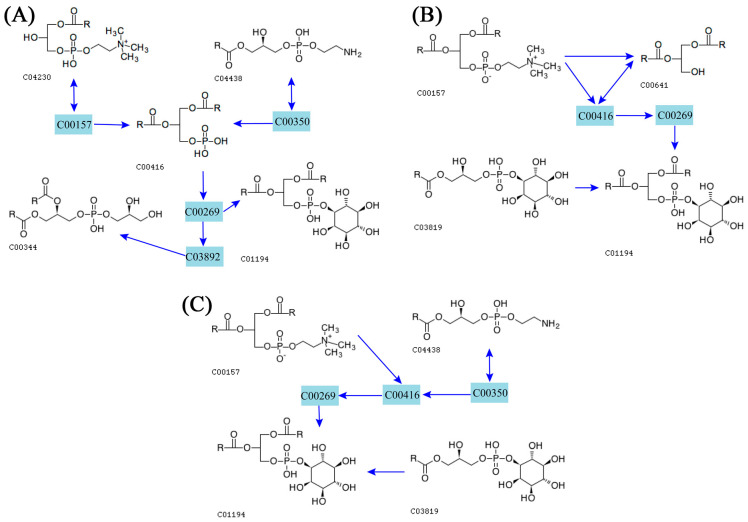
Glycerophospholipid metabolism of differential lipids in natural processing (**A**), washed processing (**B**), and honey processing (**C**) [80]. The numbers correspond to the ID of each lipid subclass.

Lipidomics has provided a more detailed view of these processing-related changes. Wang et al. [80] compared dry, wet, and semi-dry processing in Arabica coffee and identified 510 lipid metabolites across 27 subclasses. Their OPLS-DA analysis showed clear separation among processing groups, with 130, 131, and 132 differential lipids identified in the dry, wet, and semi-dry processing groups, respectively, and 114 lipids shared across all methods [80]. In total, 37 lipids were proposed as candidate markers for distinguishing processing methods. Pathway analysis further indicated that glycerophospholipid metabolism was the main pathway associated with these lipid changes (Figure 4) [80]. Collectively, these results suggest that postharvest processing reshapes the coffee lipid profile in a method-dependent manner and that glycerophospholipid remodeling may be one of the key biochemical features underlying these differences.

From an applied perspective, these findings support the use of lipidomics to evaluate and optimize coffee postharvest processing. However, most current evidence remains based on relative abundance changes and multivariate discrimination, and direct links between specific lipid markers and sensory outcomes are still limited. Future studies combining lipidomics with sensory analysis, targeted quantification, and process-standardized sampling will be necessary to establish more robust relationships between processing conditions, lipid remodeling, and coffee quality.

### 3.4. Lipidomics in Coffee Roasting

Roasting is a critical stage in coffee production, during which green beans are transformed into roasted products with characteristic aroma and flavor profiles. Typically conducted at 170–230 °C for 10–15 min, roasting induces extensive physicochemical changes, including lipid oxidation, thermal degradation, and interactions with other macromolecules [19,104,105]. These transformations are associated with the formation of volatile compounds and the modulation of sensory attributes such as aroma intensity, flavor complexity, and mouthfeel [106,107]. In addition to the final roasting degree, the time–temperature profile, heat-transfer conditions, and oxygen availability can influence lipid oxidation and thermal degradation. Higher roasting temperatures or longer roasting durations may accelerate the degradation of phospholipids and unsaturated fatty acyl chains, whereas oxygen exposure can promote the formation of oxidized lipids and lipid-derived aldehydes [81]. Because these reactions occur simultaneously with Maillard reactions and caramelization, roasting-related lipid changes should be interpreted as part of an integrated thermal reaction network rather than as isolated lipid degradation events.

Lipidomics has provided new opportunities to systematically characterize lipid transformations during roasting. Studies based on LC-MS and UPLC-ESI-MS/MS platforms have consistently shown that triacylglycerols (TAGs) remain the dominant lipid class throughout roasting, whereas phospholipids and other minor lipid components exhibit more pronounced dynamic changes [36,81]. At the subclass level, Zhu et al. [36] reported significant alterations in glycerophospholipids, including phosphatidylethanolamine (PE), phosphatidylcholine (PC), and phosphatidylinositol (PI). Within these subclasses, different annotated lipid features or putatively identified lipid species showed roasting-dependent patterns, with some decreasing progressively during roasting and others showing transient increases at early stages. These changes are distinct from conventional fatty acid compositional changes and likely reflect the combined effects of thermal degradation, oxidation, and structural rearrangement.

**Figure 5:**
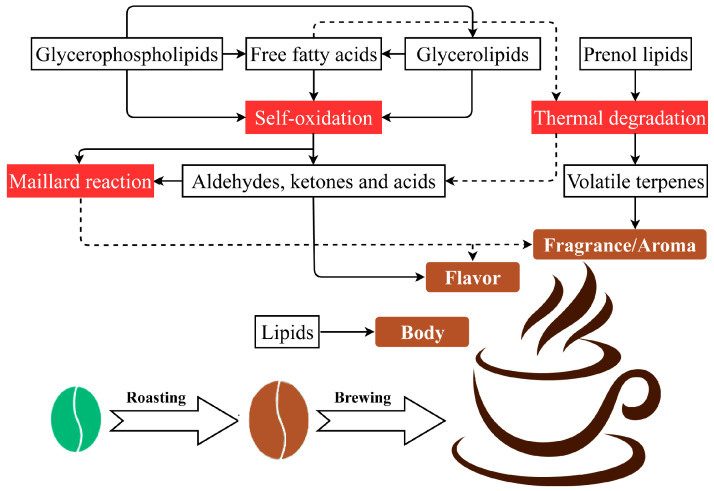
The mechanism by which coffee lipids form the sensory attributes of coffee during roasting [81].

In addition to compositional shifts, lipidomics studies have revealed that roasting leads to the generation of a wide range of lipid-derived intermediates. Oxidation of unsaturated fatty acids can produce aldehydes, ketones, and other reactive compounds, which may further participate in Maillard reactions or secondary transformations. As illustrated in Figure 5 [81], glycerophospholipids and glycerolipids can release free fatty acids under thermal conditions, which subsequently undergo oxidation and degradation to form volatile compounds contributing to aroma and flavor. However, it should be noted that most current evidence is based on compositional changes and inferred pathways, and direct causal links between specific lipid species and sensory outcomes remain limited.

Multivariate analyses, including PCA and OPLS-DA, have frequently been applied to distinguish roasting degrees based on lipid profiles, identifying sets of differential lipids associated with light, medium, and dark roasting [81]. While these approaches are useful for pattern recognition and classification, their results should be interpreted with caution, as they primarily reflect statistical associations rather than mechanistic relationships. Furthermore, differences in analytical platforms, sample preparation, and data processing strategies may influence the comparability of reported lipid markers across studies.

Collectively, existing studies suggest that lipid transformations during roasting are driven by a combination of thermal degradation and oxidative processes, leading to substantial remodeling of lipid composition and the generation of aroma-related compounds. From a practical perspective, these findings indicate that controlling roasting conditions, such as temperature profiles and duration, can influence lipid degradation pathways and thereby modulate flavor characteristics. Nevertheless, further research integrating lipidomics with sensory evaluation and targeted validation approaches is required to establish more robust links between specific lipid transformations and coffee quality.

### 3.5. Effects of Storage on Coffee Lipids

Storage is a critical factor influencing the chemical stability and sensory quality of both green and roasted coffee beans [8,82,108,109]. During storage, lipids, particularly unsaturated fatty acids, are susceptible to oxidation and hydrolysis, leading to the formation of degradation products that may affect aroma, flavor, and overall quality [82,83]. These processes are influenced by multiple factors, including temperature, relative humidity, water activity, oxygen availability, storage duration, packaging materials, and bean form [8,85,108]. Increased relative humidity and water activity may promote hydrolytic reactions, whereas elevated temperature and oxygen exposure may accelerate lipid oxidation. Packaging systems with different oxygen and moisture barrier properties, such as conventional bags, vacuum packaging, modified-atmosphere packaging, or one-way-valve packages, may therefore lead to different rates of phospholipid loss, free fatty acid accumulation, and formation of lipid-derived volatile aldehydes. From a shelf-life perspective, lipidomics may help identify early molecular indicators of quality loss, but robust links among specific lipid markers, packaging conditions, and sensory shelf-life endpoints remain insufficiently established.

Lipidomics-based studies, although still relatively limited in this area, have begun to provide insights into lipid alterations during coffee storage [84,85,86]. Existing evidence suggests that storage is generally associated with a gradual decline in certain phospholipids, such as lysophosphatidylcholine (LPC), phosphatidylcholine (PC), phosphatidylethanolamine (PE), and phosphatidylinositol (PI), alongside increases in lipid oxidation products [84]. These compositional changes are consistent with the degradation of membrane-associated lipids and the progression of oxidative reactions. At the same time, variations in total lipid content reported across studies appear to depend strongly on storage conditions, including temperature and packaging systems [85,86].

**Figure 6:**
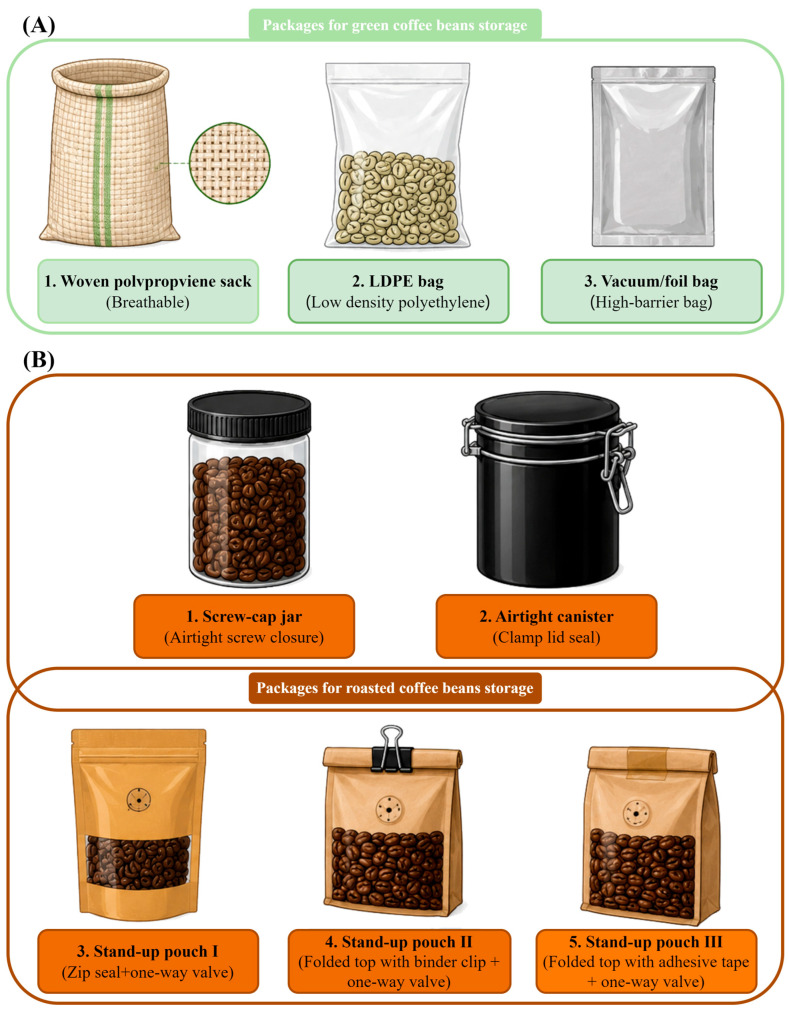
(**A**) Packages for green coffee bean storage; (**B**) packages for storing roasted coffee beans.

From a mechanistic perspective, the oxidation of unsaturated fatty acids is considered a major pathway driving lipid degradation during storage [82,83]. In a study by Cong et al. [82], the authors systematically evaluated lipid oxidation in Robusta green coffee beans during 20 days of accelerated storage at 40–60 °C. The peroxide value (a primary oxidation indicator) increased from an initial 4.20 meq/kg to 9.88 meq/kg after 20 days at 60 °C. Correspondingly, the content of linoleic acid (C18:2), the most abundant unsaturated fatty acid in coffee, decreased substantially from 354.45 mg/g oil to 28.19 mg/g oil (approximately 92% reduction), while free fatty acids increased from 1.20 to 3.33 mg KOH/g oil. These quantitative changes provide direct evidence that unsaturated fatty acid oxidation is a major driver of lipid degradation during coffee storage. This process can generate aldehydes (including hexanal, nonenal, and diene aldehydes) and other volatile compounds, which may contribute to the development of off-flavors and rancidity [83]. As summarized in Figure 6, packaging type and environmental conditions can significantly influence lipid stability by modulating oxygen exposure and moisture levels [85,110]. However, most current interpretations are based on indirect indicators, such as changes in lipid classes or conventional oxidation indices (e.g., peroxide value, free fatty acids) [69,82], and a direct linkage between specific lipid species and sensory deterioration remains insufficiently established.

In addition to compositional changes, storage studies often rely on a combination of conventional lipid oxidation parameters (e.g., AV, PV, TBARS) and lipidomics data [69,82]. While this integrative approach can provide complementary information, differences in analytical methodologies and reporting standards may limit cross-study comparability [55,57]. Furthermore, lipidomic analyses of roasted coffee during storage remain scarce, despite the higher susceptibility of thermally processed lipids to further oxidation.

Collectively, available studies indicate that lipid degradation during storage is primarily driven by oxidative processes, with environmental conditions playing a key regulatory role [82,83]. From an applied perspective, controlling storage parameters, particularly temperature, humidity, oxygen exposure, and packaging, can help mitigate lipid oxidation and preserve coffee quality [8,85,108]. Future research integrating lipidomics with sensory evaluation and targeted validation strategies is needed to better define the relationships between lipid changes and quality deterioration and to support the development of more reliable lipid-based indicators for storage stability.

## 4. Conclusions and Future Perspectives

### 4.1. Conclusions

MS-based lipidomics has become an increasingly useful approach for studying coffee lipids across the production chain. Compared with conventional lipid measurements, lipidomics provides higher molecular resolution and makes it possible to track changes in lipid subclasses and individual species during bean maturation, postharvest processing, roasting, and storage. Existing studies consistently show that coffee lipid composition is not static but is reshaped by genotype, developmental stage, processing conditions, thermal treatment, and storage environment. These changes are closely related to key quality attributes, including aroma formation, mouthfeel, oxidative stability, and product authenticity. In this sense, lipidomics has moved beyond simple compositional profiling and is beginning to contribute to a more integrated understanding of coffee quality formation. Overall, lipidomics offers a promising framework for connecting coffee composition with quality formation and stability, but its full value will depend on methodological rigor, cross-study reproducibility, and stronger links between molecular data and sensory or functional outcomes.

### 4.2. Current Limitations and Challenges

The current evidence base still has important limitations. First, most studies remain descriptive and rely heavily on relative abundance data and multivariate classification, which are useful for pattern recognition but insufficient for establishing direct mechanistic links between specific lipid species and sensory outcomes. A clear distinction should be made between well-supported findings (e.g., glycerophospholipid remodeling during maturation and roasting, species differences in triacylglycerols and diterpenes) and more preliminary observations (e.g., candidate markers for processing methods or universal maturity indicators that lack cross-validation). Second, structural annotation remains a major challenge, especially for isomeric lipids and low-abundance minor species. Third, differences in extraction protocols, analytical platforms, data processing pipelines, and reporting standards reduce cross-study comparability and make it difficult to compare candidate lipid markers across laboratories. Fourth, although several lipid species have been proposed as markers for origin, processing, or roasting degree, their robustness and reproducibility under independent validation are still limited. Fifth, systematic studies on lipid dynamics in specialty coffee resources (such as small-origin varieties, wild species, and low-caffeine beans) and in diverse product forms (including instant coffee, ready-to-drink beverages, and plant-based coffee analogs) remain limited. Similarly, lipidomic investigations of coffee storage are scarce. Furthermore, it is important to acknowledge that most links between lipid transformations and specific flavor attributes remain indirect. Few studies have directly traced a lipid-derived volatile from precursor to final cup or validated its sensory contribution via recombination experiments. The current evidence is thus largely correlational rather than causal.

### 4.3. Future Perspectives

Despite these gaps, several lipid classes stand out as particularly promising for practical quality control applications. Glycerophospholipids (especially PC, PE, and their lyso-forms) are sensitive indicators of processing and storage freshness. Diterpenes (cafestol, kahweol, and 16-O-methylcafestol) serve as robust markers for species authentication and green bean origin. Triacylglycerol profiles reflect genetic and environmental backgrounds, offering utility for traceability. Oxidized lipid-derived volatiles (e.g., hexanal, nonenal) show potential for monitoring storage-induced quality loss. For routine industrial applications, a tiered strategy is recommended: rapid NIR screening calibrated against lipidomics data, followed by targeted LC-MS or GC-MS for confirmation of key markers.

For practical implementation in coffee quality-control laboratories, cost and infrastructure should be considered together with analytical performance. High-resolution untargeted lipidomics is valuable for marker discovery, but its instrument cost, data-processing burden, and requirement for specialized personnel limit routine use. In contrast, targeted LC-MS/MS or GC-MS methods based on a small panel of validated markers may be more suitable for regular quality-control testing. Such assays should include appropriate internal standards, preferably stable isotope-labeled standards when available or class-specific surrogate standards for TAGs, phospholipids, free fatty acids, diterpenes, and lipid-derived oxidation products. Before routine application, candidate marker panels should be evaluated through matrix-matched calibration, pooled quality-control samples, recovery assessment, repeatability testing, and interlaboratory validation. In actual laboratory use, lipidomics-based methods are most likely to serve as confirmatory tools for species authentication, roasting-degree assessment, oxidation monitoring, storage stability evaluation, and shelf-life investigation rather than as stand-alone replacements for conventional sensory and physicochemical analyses.

Future research should therefore move from broad profiling toward more standardized, validation-oriented designs. Priority areas include improved structural elucidation using high-resolution mass spectrometry, ion mobility, and orthogonal fragmentation strategies; targeted quantification of candidate markers with stable isotope standards; and integration of lipidomics with sensory evaluation, metabolomics, and process parameters. In particular, studies that combine lipidomic profiling with controlled experiments are needed to distinguish true process-driven lipid remodeling from variation caused by genotype or environment. For coffee storage, more work is needed on roasted beans, where lipid oxidation may be especially relevant to quality loss. For coffee processing and roasting, future studies should focus not only on identifying differential lipids but also on clarifying which lipid changes are most directly linked to desirable or undesirable sensory attributes. A more standardized and mechanism-oriented research agenda will be essential for translating lipidomics from descriptive profiling into practical tools for coffee quality control, traceability, and process optimization.

## Figures and Tables

**Table 1:** Summary of major coffee lipidomics studies and evidence strength assessment.

Research Area	Key Findings	Evidence Strength	Main Limitations	References
Maturation	Glycerophospholipids and glycerolipids, including PA, LPA, and DG, are remodeled during fruit development.	Moderate	Direct links to sensory outcomes have not been established; maturity definitions vary across studies.	[65,66]
	Specific lipid species have been proposed as candidate maturity markers.	Preliminary	Independent validation across diverse genotypes, environments, and harvest years is still needed.	[65,66]
Species/variety discrimination	Arabica and Robusta differ in TAGs, fatty acids, diterpenes, sterols, and tocopherols.	Relatively strong	Environmental effects may confound species-specific signals; universal cut-off values are not yet available.	[34,67,68,69,70,71,72,73,74,75,76]
	Lipid profiles can separate cultivars or populations within Arabica.	Moderate	Larger multi-origin validation is required; maturity stage and processing method may influence classification.	[77,78,79]
Postharvest processing	Glycerophospholipid metabolism appears to be a major pathway affected by dry, wet, and semi-dry processing; 37 candidate markers have been proposed.	Moderate	Sensory correlation was not performed; candidate markers require validation across coffee types, regions, and harvest years.	[80]
Roasting	Phospholipids, including PC, PE, and PI, change markedly with roasting degree.	Moderate	The degradation mechanisms and their contribution to specific aroma compounds remain incompletely elucidated.	[36,81]
	Oxidized lipid species and volatile aldehydes increase during roasting.	Moderate	Direct precursor-product tracking from specific lipid species to defined flavor notes is still lacking.	[36,81]
	Specific TAGs or lipid ratios may serve as candidate markers of roasting degree.	Preliminary to moderate	Targeted quantification and validation across different roasting profiles are needed.	[36,81]
Storage	Unsaturated fatty acids undergo oxidation during storage, generating aldehydes such as hexanal and nonenal that may contribute to off-flavors.	Moderate	Sensory threshold levels and their matrix-dependent effects in coffee have not been fully determined.	[82,83]
	Phospholipids, including LPC, PC, PE, and PI, decline during storage.	Moderate	Changes are strongly dependent on packaging, temperature, humidity, oxygen exposure, and storage duration; no universal storage marker has been established.	[84]
	Lipidomics may support early detection of coffee quality loss during storage.	Preliminary	Roasted coffee storage remains under-studied; stronger correlation with sensory shelf-life is needed.	[84,85,86]

Note: TAGs, triacylglycerols; PA, phosphatidic acid; LPA, lysophosphatidic acid; DG, diacylglycerol; PC, phosphatidylcholine; PE, phosphatidylethanolamine; PI, phosphatidylinositol; LPC, lysophosphatidylcholine. Evidence strength was qualitatively assigned based on the consistency of reported findings, number of studies, degree of validation, and availability of mechanistic or sensory support.

## Data Availability

No new data were created or analyzed in this study. Data sharing is not applicable to this article.

## Abbreviations

The following abbreviations are used in this manuscript: ANOVA, analysis of variance; APCI, atmospheric pressure chemical ionization; AV, acid value; CAD, charged aerosol detector; DART, direct analysis in real time; DG, diacylglycerol; DI-MS, direct infusion mass spectrometry; DLLE, dispersive liquid–liquid microextraction; ESI, electrospray ionization; FLD, fluorescence detector; FT-ICR, Fourier-transform ion cyclotron resonance; FTIR, Fourier-transform infrared; GA, genetic algorithm; GC, gas chromatography; GC-MS, gas chromatography–mass spectrometry; HCA, hierarchical cluster analysis; ILME, ionic liquid microextraction; IM-MS, ion mobility–mass spectrometry; LC, liquid chromatography; LC-MS, liquid chromatography–mass spectrometry; LDA, linear discriminant analysis; LPA, lysophosphatidic acid; LPC, lysophosphatidylcholine; MAE, microwave-assisted extraction; MLR, multiple linear regression; MS, mass spectrometry; MSI, mass spectrometry imaging; MTBE, methyl tert-butyl ether; MVDA, multivariate data analysis; NIR, near-infrared; OPLS-DA, orthogonal partial least squares discriminant analysis; PA, phosphatidic acid; PC, phosphatidylcholine; PCA, principal component analysis; PE, phosphatidylethanolamine; PI, phosphatidylinositol; PLS, partial least squares regression; PLS-DA, partial least squares discriminant analysis; PV, peroxide value; QQQ, triple quadrupole; QTOF, quadrupole time of flight; RF, random forest; SPE, solid-phase extraction; SFE, supercritical fluid extraction; SPME, solid-phase microextraction; SVM, support vector machine; TAG, triacylglycerol; TBARS, thiobarbituric acid reactive substances; TOF, time of flight; UAE, ultrasonic-assisted extraction; UHPLC, ultra-high performance liquid chromatography.
